# A combined histo-score based on tumor differentiation and lymphocytic infiltrate is a robust prognostic marker for mobile tongue cancer

**DOI:** 10.1007/s00428-020-02875-9

**Published:** 2020-06-30

**Authors:** Inger-Heidi Bjerkli, Elin Hadler-Olsen, Elisabeth Sivy Nginamau, Helene Laurvik, Tine M. Søland, Daniela Elena Costea, Lars Uhlin-Hansen, Sonja E. Steigen

**Affiliations:** 1 Department of Otorhinolaryngology, University Hospital of North Norway, Tromsø, Norway; 2 Department of Medical Biology, UiT The Arctic University of Norway, Tromsø, Norway; 3 The Public Dental Health Service Competence Center of Northern Norway, Tromsø, Norway; 4 Department of Pathology, Haukeland University Hospital, Bergen, Norway; 5 Center for Cancer Biomarkers CCBIO and The Gade Laboratory of Pathology, Department of Clinical Medicine, University of Bergen, Bergen, Norway; 6 Department of Pathology, Rikshospitalet, Oslo University Hospital, Oslo, Norway; 7 Institute of Oral Biology, Faculty of Dentistry, University of Oslo, Oslo, Norway; 8 Department of Pathology, University Hospital of North Norway, Tromsø, Norway

**Keywords:** Oral squamous cell carcinoma, Low-stage, Differentiation, Lymphocyte infiltrate, Histo-score, Prognostic factors

## Abstract

We wanted to evaluate the prognostic value of common histopathological variables in a large cohort of patients with cancer in the mobile tongue as such information can be important for treatment stratification of the individual patient, especially for patients with low-stage disease. In addition, we wanted to investigate whether an alternative scoring model with fewer options would compromise the prognostic value. One hundred fifty patients with oral tongue squamous cell carcinomas that were treated in curative intent and with available HE-stained tumor sections were included. We reclassified all tumors and performed univariate and multivariate survival analyses of histopathological and clinical variables. For the complete cohort, lymph node status, grade of differentiation, perineural infiltration, and lymphocytic infiltration were independent prognosticators. In the low-stage disease group, independent prognostic factors were tumor size, grade of differentiation, and lymphocytic infiltrate. For patients with low-stage disease, a histo-score combining the scores for tumor differentiation and lymphocytic infiltrate identified a group of patients with particularly low survival, as patients with moderately or poorly differentiated tumors and little lymphocytic infiltrate had a less favorable 5-year survival outcome than patients in the high-stage disease group. This study shows that a histo-score combining tumor differentiation and lymphocytic infiltration should be given special consideration in treatment planning. Our results also illustrate that many variables can be scored with fewer options than previously suggested to increase their reproducibility, and still maintain their prognostic value.

**Electronic supplementary material:**

The online version of this article (10.1007/s00428-020-02875-9) contains supplementary material, which is available to authorized users.

## Introduction

About half of all malignant tumors in the oral cavity arise in the mobile, anterior two-thirds of the tongue, and more than 90% of them are squamous cell carcinomas (SCC) [1]. The aggressiveness of oral cavity tongue (OT) SCC varies markedly, even for small tumors without lymph node metastases [2]. The search for morphological tumor traits that reliably predict the prognosis for the individual patient has been going on for decades [3–5]. Such prognostic markers could help clinicians select the optimal treatment for individual patients that could increase the chances of being cured of the disease, and at the same time minimize the side effects from overtreatment. The TNM system classifies tumors based on their size and depth of invasion (T), neck node involvement (N), and distant metastasis (M). Along with the International Union against cancer (UICC) staging, these factors are today the best survival prognosticators for cancers in the oral cavity [6]. On the group level, patients with low-stage disease (stages I–II; T1–2, N0M0) have an estimated higher survival rate compared with patients with high-stage disease (stages III–IV) [7, 8]. However, there is a need to find markers that can differentiate between aggressive and more indolent tumors for individual patients within the same stage.

Various aspects of a tumor’s morphology and growth pattern can be evaluated on hematoxylin and eosin (HE)-stained tumor sections. Several of these characteristics have been proposed as prognostic markers in oral cancer [4, 9, 10]. However, despite some reports of prognostic usefulness, none of these markers has been implemented in clinical practice, mostly due to lack of coherence between studies. There are several putative explanations for the lack of consistency between prognostic studies. Many are based on small patient cohorts and do not control for parameters known to affect prognosis, such as intraoral location and stage [11–14]. This biases the actual prognostic value of the markers in question. Furthermore, the evaluation of histopathological criteria is subjective, and different pathologists may interpret the same criterion differently [15, 16]. In a recent study, we found poor inter- and intra-observer agreement when evaluating a selection of proposed histopathological prognostic markers in oral SCC, even though the observers had mutual training sessions and were experienced pathologists [17]. Improved agreement was obtained by reducing the number of scoring alternatives for each parameter. This suggests that fewer options for each parameter might increase the robustness of histopathological prognostic markers, provided that the reduction of scoring alternatives does not compromise the prognostic value. In the current study, we evaluated the prognostic value of a number of proposed histopathological variables as they were originally proposed, as well as with a reduced number of scoring alternatives, in a large, homogenous cohort of OTSCC. Our results show that some histopathological markers, individually and in combination, can add significant prognostic information for OTSCC. Our study further highlights the importance of controlling for known risk factors such as tumor size and lymph node metastasis when evaluating putative prognostic markers.

## Materials and methods

### Cohort of patients

The Norwegian Oral Cancer (NOROC) study is a retrospective study that includes patients diagnosed with oral cavity SCC in Norway from January 1, 2005, through December 31, 2009.The NOROC study includes patients with strict oral cavity SCC [8]. In the present study, the relevant ICD-10 codes were C02, which refer to cancers in the mobile tongue. Of the original NOROC cohort, 273 patients (45%) had OTSCC. From them, we included only the primary, treatment-naïve patients who were treated in curative intent and from whom we had HE-stained sections from biopsies or resections available, altogether 150 patients.

### Extracting clinical and histopathological data

Experienced head and neck surgeons retrieved clinical parameters from the electronic health records as previously described [8]. Of the 150 patients that underwent surgery, 72 patients had neck surgery, and for them, the N-status was based on histopathological evaluation. For the patients who did not have neck surgery, the N-status was based on clinical/radiological evaluation.

Senior pathologists re-evaluated the histopathological characteristics of the tumors, including WHO degree of differentiation, keratinization, nuclear polymorphism, perineural infiltration, lymphocyte infiltrate at the interface between tumor and surrounding stroma, and worst pattern of invasion [3, 4]. For several of these, a fairly elaborate grading system was originally suggested. In this study, we have also applied alternative versions, as described in our previous paper [17] and summarized in Table 1. The pathologists were blinded for the patients’ clinical information and outcome.

**Table 1:** Variables with original and alternative grading

	Tumor characteristics	Original variables	Alternative 1		Alternative 2	
1.0	Differentiation whole tumor	Well	1.1	Low-grade		
		Moderate				
		Poorly		High-grade		
2.0	Differentiation worst pattern	Well	2.1	Low-grade		
		Moderate				
		Poorly		High-grade		
3.0	Keratinization whole tumor	High	3.1	Low-grade		
		Moderate				
		Minimal		High-grade		
		None				
4.0	Keratinization tumor front*	High	4.1	Low-grade		
		Moderate				
		Minimal		High-grade		
		None				
5.0	Polymorphism whole tumor	Little/none	5.1	Low-grade		
		Moderate				
		Abundant		High-grade		
		Extreme				
6.0	Polymorphism tumor front*	Little/none	6.1	Low-grade		
		Moderate				
		Abundant		High-grade		
		Extreme				
7.0	Perineural infiltration	None	7.1	No		
		Invasive front		Yes		
		Tumor center				
8.0	Lymphocyte infiltrate	Marked	8.1	Marked	8.2	Abundant
		Moderate		Not marked		
		Little/none				Little
9.0	Worst pattern of invasion (WPO)**	Type 1	9.1	Low-grade	9.2	Low-grade
		Type 2				
		Type 3		High-grade		
		Type 4				High-grade
		Type 5				

*According to Bryne et al. [3]

**According to Brandwein-Gensler et al. [4]. Type 1 pushing borders; Type 2 finger-like growth pattern; Type 3 large separate islands, > 15 cells per island; Type 4 small tumor islands, ≤ 15 cells per island; Type 5 tumor satellites, ≥ 1 mm from main tumor/satellite

We calculated survival from the date of diagnosis until the date of death or last day of follow-up, which was June 1, 2015. At that time, all patients were followed up for a minimum of 5 years or until death. Cause of death was acquired from the Norwegian Cause of Death Registry.

The study was approved by the Northern Norwegian Regional Committee for Medical Research Ethics (Protocol numbers REK Nord; 2013/1786 and 2015/1381). Patients still alive were informed about the project and had the opportunity to opt-out.

### Statistical analysis

Descriptive analyses and univariate survival analyses using log-rank (Mantel-Cox) giving Kaplan-Meier survival curves were performed. Variables significant in univariate calculations were tested for collinearity before entering them into multivariable equations. Multivariate survival analyses were performed using Cox regression model. Associations were investigated using chi-square. Receiver operating characteristic curve was applied to evaluate cut-off values in binary classifications. All statistical analyses were performed using SPSS version 26. All survival analyses were significant at 0.05 level.

## Results

One hundred and fifty patients with OTSCC were eligible for histopathological reclassification and included in the study. Of the tumor material available, 127 were resection specimens, 18 biopsies, and 5 unknown. Seventy-seven patients had low-stage disease (stages I and II according to TNM 8th edition), 63 had high-stage (stages III and IV) [6], and for 10 cases the information for stage was missing.

Supplementary Table 1 presents the scores for each variable for the whole cohort and after separation into low-stage and high-stage disease. Table 2 presents gender, age, TNM-status and stage, as well as calculation of 5-year disease-specific survival (DSS).

**Table 2:** Clinicopathological characteristics related to 5-year disease specific survival (DSS). Number and percent of patients in each group, and in addition percentage of patients with 5-year DSS

Variable		*n*	%	DSS %	*p* *(DSS)
Gender	Male	92	61.3	70.7	0.702
	Female	58	38.7	69.0	
Age (year) group	≤ 50	29	19.3	79.3	0.015
	51–60	29	19.3	65.5	
	61–70	44	29.3	70.5	
	71–80	31	20.7	67.7	
	≥ 81	17	11.3	64.7	
pT 8th Edition	pT1	48	35.6	87.5	0.006
	pT2	53	39.3	64.2	
	pT3	34	25.2	64.7	
N**	N0	108	72.0	82.4	0.001
	N+	40	26.7	37.5	
	Nx/Unknown	2	1.3		
Stage	Low stage	77	55.0	82.8	< 0.001
	High stage	63	42.0	44.6	
	Nx/Unknown	10	6.7		

*Significant at 0.05 level

**Combination of cN and pN. If neck dissection was performed the result on pN was superior to cN

### Survival

The 5-year DSS was 64.8% for the whole group and 82.8% and 44.6% for the low- and high-stage group, respectively.

#### Univariate analyses

In Table 3, 5-year DSS from univariate analyses are listed for each variable, both with original and alternative versions of grading. For the whole cohort, the following variables were significantly associated with 5-year DSS: degree of differentiation (1.0 and 1.1), keratinization of the whole tumor (3.0 and 3.1), keratinization at tumor front (4.0 and 4.1), perineural infiltration (7.0 and 7.1), lymphocytic infiltrate (8.0, 8.1, and 8.2), and worst pattern of infiltration (9.2).

**Table 3:** Variables (both original and alternative grading) and 5-year disease-specific survival in univariate calculations for the whole cohort, and for low-stage disease and high-stage disease separately. The percentage of patients surviving according to different grading is specified under DSS%

		Whole cohort (*n* = 150)		Low-stage (*n* = 78)		High-stage (*n* = 63)	
	Variables		All		All		All
		DSS %	*p* (*n*)	DSS%	*p* (*n*)	DSS%	*p* (*n*)
1.0	Differentiation, whole tumor	92.3/64.0/16.7	< 0.001 (127)*	94.7/84.6/40.0	0.002 (63)*	75.0/47.7/0	0.055 (55)
	1.1	70.4/16.7	< 0.001*	87.7/40.0	0.001*	51.1/0	0.025 *
2.0	Differentiation, worst pattern	100/72.0/56.7	0.074 (124)	100/88.9/76.9	0.248 (63)	100/50.0/40.0	0.646 (54)
	2.1	75.4/56.7	0.053	90.9/76.7	0.121	52.6/40.0	0.676
3.0	Keratinization whole tumor	85.0/68.0/53.6/47.6	0.038 (124)*	82.4/91.3/83.3/66.7	0.402 (61)	83.3/46.2/30.8/22.2	0.137 (54)
	3.1	73.3/51.0	0.010 *	87.5/76.2	0.238	53.1/27.3	0.078
4.0	Keratinization, tumor front	/100/70.3/59.7	0.047 (120)^*^	÷/100/89.5/75.8	0.133 (62)	÷/100/47.1/44.1	0.665 (52)
	4.1	100/63.3	0.025*	100/80.8	0.146	100/45.1	0.374
5.0	Nuclear polymorphism	84.0/64.4/60.0/52.0	0.147(125)	100/85.7/84.6/50.0	0.004 (62)*	33.3/42.1/40.0/50.0	0.767 (54)
	5.1	71.4/56.4	0.097	92.3/69.6	0.018*	40.0/44.8	0.673
6.0	Nuclear polymorphism, tumor front	66.7/75.0/68.8/57.5	0.507 (120)	100/94.7/64.2/64.7	0.054 (63)	20.0/46.7/40.0/54.5	0.363 (52)
	6.1	72.9/62.5	0.302	96.2/75.0	0.029*	40.0/50.0	0.343
7.0	Perineural infiltration	70.0/52.9/28.6	0.003 (114)*	84.0/71.4/50.0	0.250 (59)	52.8/33.3/20.0	0.046 (50)*
	7.1	70.0/45.8	0.020*	84.0/66.7	0.208	52.8/28.6	0.098
8.0	Lymphocytic infiltration	87.5/67.2/50.0	0.008 (123)*	100/87.5/56.3	0.003 (63)*	62.5/40.0/47.6	0.711 (54)
	8.1	87.5/60.0	0.021*	100/77.1	0.050*	62.5/43.5	0.409
	8.2	72.9/50.0	0.007*	91.5/56.3	0.001*	45.5/47.6	0.821
9.0	WPOI	100/70.6/82.8/58.9/56.3	0.190 (120)	100/80.0/100/77.8/66.7	0.224 (62)	÷/50.0/50.0/42.3/50.0	0.997 (52)
	9.1	73.7/65.3	0.461	83.3/84.0	0.999	50,0/45.7	0.908
	9.2	79.2/58.3	0.022*	93.1/75.8	0.061	50.0/44.4	0.902

*Significant at 0.05 level

For patients with low-stage disease, differentiation of the whole tumor (1.0 and 1.1), nuclear polymorphism whole tumor (5.0 and 5.1), nuclear polymorphism at tumor front (6.1), and lymphocytic infiltrate (8.0, 8.1, and 8.2) were significantly associated with DSS. For patients with high-stage disease, differentiation of whole tumor (1.1) and perineural infiltration (7.0) were the only significant prognosticators of DSS.

Separate calculations were also performed for resection specimens only (biopsies excluded) with results similar to those for all tumors (resections and biopsies), and it is presented in Supplementary Table 2*.*

#### Multivariate analyses

We performed multivariate analysis of the histopathological variables that were significant in univariate calculations, with separate analyses for original and alternative grading of the variables. Additionally, T and lymph node status was included in the equation for the whole cohort, and T for the low-stage disease group. For the whole cohort, this included differentiation, keratinization, perineural infiltration, and lymphocytic infiltration for both original and alternative scoring gradings, and WPOI was additionally included in the alternative version. Keratinization of the whole tumor and keratinization of the tumor front were collinear, and only keratinization of the whole tumor (3.0/3.1) was included in multivariate analyses. For patients with low-stage disease differentiation, nuclear polymorphism and lymphocytic infiltration were included. Nuclear polymorphism of the whole tumor and in the tumor front were collinear, and only polymorphism of whole tumor (5.0/5.1) was included in the multivariate analyses. Independent prognosticators for the complete patient cohort were lymph node status (*N*, *p* < 0.001), differentiation of whole tumor (1.1, *p* = 0.022), perineural infiltration (7.0, *p* = 0.025), and lymphocytic infiltration (8.2, *p* = 0.048). In the low-stage group, T (*p* = 0.003), differentiation of whole tumor (1.1, *p* = 0.022), and lymphocytic infiltrate (8.2, *p* = 0.003) were all independent variables.

#### Combined histo-score

**Fig. 1:**
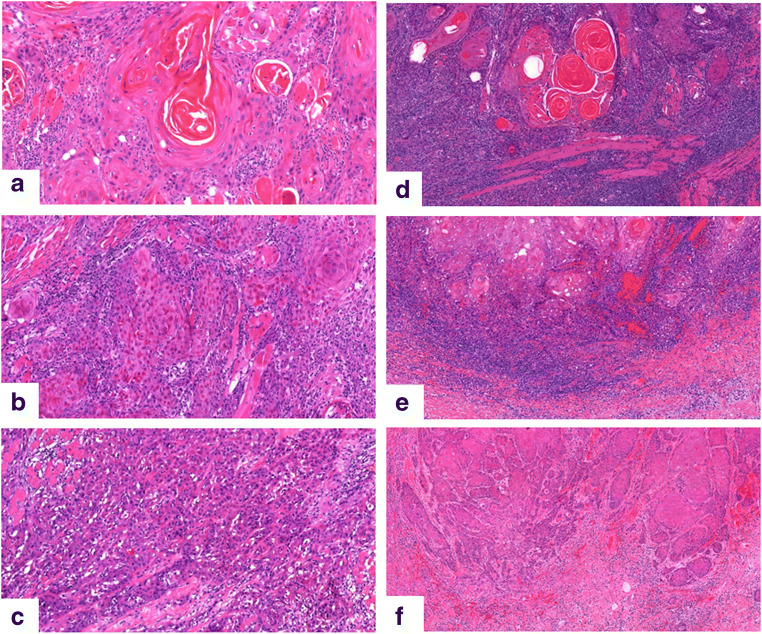
Tumor differentiation and lymphocyte infiltration. Well, moderate, and poorly differentiated tumor in **a**–**c**. Marked, moderate, and little lymphocyte infiltration in **d**–**f**

**Fig. 2:**
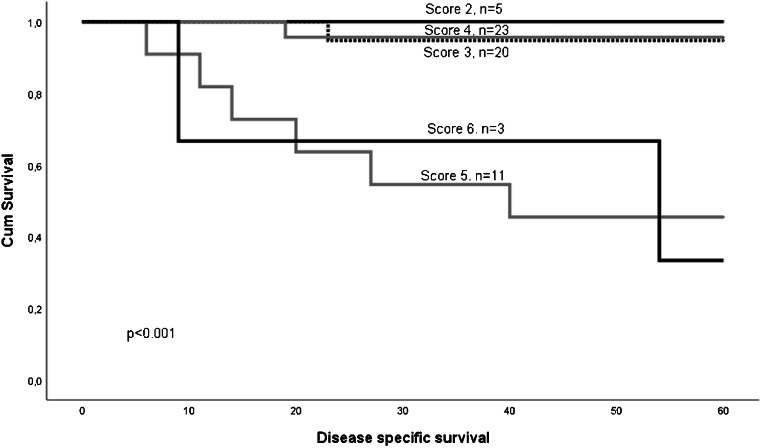
Kaplan-Meier curve showing the results after combining the variables of differentiation (1.0) and lymphocytic infiltrate (8.0). Scoring alternatives are shown in Table 3. Patients with low score (2–4) had a statistically better survival than those with high score (5–6). Figure 2 Kaplan-Meier curve showing survival of patients with low-stage disease stratified according to the histo-score

For the low-stage group, we created a combined score, called histo-score, based on tumor differentiation and lymphocytic infiltrate (Fig. 1). The histo-score was calculated by summarizing the individual score of differentiation and lymphocyte infiltration (Supplementary Table 3). Using the original grading, the lowest score was 2 and the highest was 6. There was a highly significant difference in survival between the groups (*p* < 001), Fig. 2. Of the 48 patients with scores 2, 3, or 4, only two patients died of the disease within 5 years (DSS = 95.8%). Of the 14 patients with a score of 5 or 6, eight patients died within 5 years (DSS 42.9%). The area under the ROC curve was estimated to be 0.748. The combined histo-score based on the alternative grading differentiation 1.1 and lymphocytic infiltration 8.2 showed the same significant prognostic power (*p* < 0.001).

There were no common denominators for the patients with low-stage disease and low versus high histo-score who died with respect to age, gender, T-stage, keratinization, or worst pattern of infiltration. Additionally, we explored whether there was a difference between different treatment options (with or without neck dissection, with or without postsurgical radiotherapy), but we could not find any associations.

## Discussion

Reliable, prognostic markers that can supplement tumor staging are lacking for oral cavity cancer. As tumors of the same stage can have different degrees of aggressiveness, there is a need to find additional markers to assist the treatment planning and to predict the outcome of individual patients. Oral cancer is most prevalent in developing countries [18]. Thus, markers that do not require expensive equipment or reagents, such as histopathological traits that can be assessed on HE-stained sections, are especially valuable. In the present study, we have evaluated the prognostic power of a number of histopathological variables suggested for oral cancer, where results from previous studies are contradictory [19, 20]. We have tested them in a large, homogenous cohort of patients with OTSCC, in which clinical and histopathological parameters are well controlled. Our hypothesis was that the lack of consistency of prognostic value in previous studies can be partly explained by small cohorts of patients and the inclusion of tumors from various intraoral locations. Furthermore, scoring of histopathological parameters is subjective, as reflected by the poor inter- and intra-rater agreement [17]. Several of the histological variables have been proposed with three to six options for scoring, sometimes with subtle differences between each alternative. Grouping categories and thereby reducing the number of scoring alternatives can make the scoring easier and more reproducible [17]. Therefore, we also tested the prognostic power of the variables as they were originally proposed, as well as with broader and fewer categories.

As expected, the well-established prognostic markers’ tumor size and lymph node metastases were independent predictors of survival. Additionally, we found that tumor differentiation was an independent prognosticator of survival both for the whole cohort and for patients with low-stage disease. WHO lists differentiation as a prognostic marker for oral cavity cancer, and the degree of differentiation is usually described in pathology reports [21]. However, due to many studies reporting low prognostic value of differentiation for oral SCC, clinicians rarely give it much emphasis during treatment planning [22, 23]. Our results indicate that this marker has significant prognostic power.

A revised grading for lymphocytic infiltration where the categories for marked and moderate infiltration were combined was also an independent prognostic marker for low-stage disease and for the whole patient cohort. Grouping categories generates larger groups for statistical analyses, and this can affect the significance level. However, the cutoff for dichotomizing the original three-tier variable was important. The variable only had independent prognostic power when separating the tumors with low lymphocytic infiltration from those with moderate and abundant infiltration. This suggests that lymphocyte infiltration is tightly related to the biology of the tumor and that the tumors with little infiltration may take a more aggressive course. This is in line with previous studies showing that a rich lymphocyte infiltration is associated with favorable prognosis [24–26].

By incorporating the degree of differentiation and lymphocytic infiltration in a combined histo-score, we were able to define a subgroup of patients with low-stage disease that had a much lower survival rate than the rest of the low-stage disease patients. Interestingly, the survival in this subgroup was even less favorable than patients with high-stage disease (42.9 versus 46.6%). This indicates that patients with poorly differentiated tumors with a weak lymphocytic response should be regarded as high-risk patients who need special attention, even if the tumors are small and without lymph node metastases.

Perineural infiltration was a significant prognostic marker for the whole cohort and for patients with high-stage disease, but not for low-stage disease. One could assume that nerve bundles are more abundant in deeper parts of the oral mucosa, and tumors probably need to invade deeper than in T1 and T2 tumors for this to be a relevant marker. This illustrates the importance of evaluating prognostic markers in homogenous groups of tumors and controlling for known risk factors.

We found that alternative grading (fewer options) of histopathological variables only altered their prognostic value only to a minor extent. A previous study comparing inter- and intra-rater agreement showed significantly better agreement when using an alternative grading with fewer options, compared to the more elaborate original grading [17]. This supports the use of variables with fewer options as they improve the reproducibility of the scoring without reducing the prognostic power of the variables. A simplification of scoring models has been introduced for many cancers. The reproducibility for uterine endometrial endometrioid carcinoma was found to be higher with a binary tumor grading system [27]. In the latest WHO-classification of tumors in the GI tract, the adenocarcinomas are stratified into a two-tiered grading system, low-grade and high-grade, where grading is based on the least differentiated component [28].

The present study is retrospective, and this approach gives a larger risk of variation in how clinical variables are reported in the electronic health records compared with prospective studies. When subgrouping, some groups became small, which increases the risk of underpowered statistical analyses and thereby underestimating the prognostic power of some variables. Our cohort included some tumors from which we had only biopsy samples for histopathological evaluation, which makes evaluation less certain. Therefore, we performed separate statistical analyses excluding the grading of biopsies (supplementary tables), but this did not alter the results significantly.

## Conclusion

Our study on a large, homogenous tumor cohort of OTSCC shows that a histo-score combining tumor differentiation and lymphocytic infiltration identified a subgroup among the low-stage disease patients that had lower DSS than the average patients with high-stage disease. This subgroup should be given special consideration in treatment planning. Our results also illustrate that many variables can be scored with fewer options than previously suggested to increase their reproducibility, and still maintain their prognostic value.

## Electronic supplementary material

ESM 1 (SAV 12 kb)

ESM 2 (DOCX 23 kb)

ESM 3 (DOCX 19 kb)

ESM 4 (DOCX 17 kb)

## Data Availability

Statistics file with generated data is provided.
