# Supplementary material for: A combined histo-score based on tumor differentiation and lymphocytic infiltrate is a robust prognostic marker for mobile tongue cancer
Source: Virchows Arch. 2020 Jun 30;477(6):865–72. doi: 10.1007/s00428-020-02875-9 (PMC7683438; doi:10.1007/s00428-020-02875-9)
Supplement: Supplementary file 2 — (DOCX 23 kb) [file 428_2020_2875_MOESM2_ESM.docx]

| **Supplementary table S1. Histopathological information for the whole cohort (n=150), the low-stage (n=77) and the high-stage group (n=63)** | | | | | | | |
| --- | --- | --- | --- | --- | --- | --- | --- |
|  |  | **Whole tumor** | | **Low-stage** | | **High-stage** | |
| **Variable** |  | **n** | **%** | **n** | **%** | **n** | **%** |
| Differentiation whole tumor | Well | 34 | 22.7 | 26 | 33.8 | 5 | 7.9 |
|  | Moderate | 100 | 6.7 | 45 | 58.4 | 48 | 76.2 |
|  | Poorly | 14 | 9.3 | 5 | 6.5 | 9 | 14.3 |
|  | Missing/Not evaluable | 2 | 1.3 | 1 | 1.3 | 1 | 1.6 |
|  |  |  |  |  |  |  |  |
| Differentiation worst pattern | Well | 10 | 6.7 | 9 | 11.7 | 1 | 1.6 |
|  | Moderate | 62 | 41.3 | 36 | 46.8 | 21 | 33.3 |
|  | Poorly | 73 | 48.7 | 31 | 40.3 | 39 | 61.9 |
|  | Missing/Not evaluable | 5 | 3.3 | 1 | 1.3 | 2 | 3.2 |
|  |  |  |  |  |  |  |  |
| Keratinization whole tumor | High | 33 | 22 | 23 | 29.9 | 8 | 12.7 |
|  | Moderate | 60 | 40 | 29 | 37.7 | 30 | 47.6 |
|  | Minimal | 29 | 19.3 | 12 | 15.6 | 13 | 20.6 |
|  | None | 23 | 15.3 | 10 | 13.0 | 10 | 15.9 |
|  | Missing/Not evaluable | 5 | 3.3 | 3 | 3.9 | 2 | 3.3 |
|  |  |  |  |  |  |  |  |
| Keratinization tumor front* | High | 0 | 0 | 0 | 0 | 0 | 0 |
|  | Moderate | 15 | 10 | 14 | 18.7 | 1 | 1.6 |
|  | Minimal | 41 | 27.3 | 23 | 29.9 | 17 | 27.0 |
|  | None | 85 | 56.7 | 38 | 49.4 | 41 | 65.1 |
|  | Missing/Not evaluable | 9 | 6 | 2 | 2.6 | 4 | 6.3 |
|  |  |  |  |  |  |  |  |
| Nuclear polymorphism whole tumor | Little/none | 31 | 20.7 | 23 | 29.9 | 7 | 11.1 |
|  | Moderate | 51 | 34 | 26 | 33.8 | 19 | 30.2 |
|  | Abundant | 35 | 23.3 | 15 | 19.5 | 18 | 28.6 |
|  | Extreme | 29 | 19.3 | 11 | 14.3 | 17 | 27.9 |
|  | Missing/Not evaluable | 4 | 2.7 | 2 | 2.6 | 2 | 3.2 |
|  |  |  |  |  |  |  |  |
| Nuclear polymorphism tumor front* | Little/none | 14 | 9.3 | 9 | 11.7 | 5 | 7.9 |
|  | Moderate | 43 | 28.7 | 25 | 32.5 | 16 | 25.4 |
|  | Abundant | 36 | 24 | 21 | 27.3 | 11 | 17.5 |
|  | Extreme | 48 | 32 | 20 | 26.0 | 27 | 42.9 |
|  | Missing/Not evaluable | 9 | 6 | 2 | 2.6 | 4 | 6.3 |
|  |  |  |  |  |  |  |  |
| Perineural infiltration | None | 106 | 70.7 | 60 | 77.9 | 42 | 66.7 |
|  | Invasive front | 21 | 14 | 9 | 11.7 | 10 | 15.9 |
|  | Tumor center | 7 | 4.7 | 2 | 2.6 | 5 | 7.9 |
|  | Missing/Not evaluable | 16 | 10.7 | 6 | 7.8 | 6 | 9.5 |
|  |  |  |  |  |  |  |  |
| Lymfocytic infiltrate | Marked | 30 | 20 | 21 | 27.3 | 8 | 12.7 |
|  | Moderate | 67 | 44.7 | 34 | 44.2 | 28 | 44.4 |
|  | Slight/none | 46 | 30.7 | 21 | 27.3 | 24 | 38.1 |
|  | Missing/Not evaluable | 7 | 4.7 | 1 | 1.3 | 3 | 4.8 |
|  |  |  |  |  |  |  |  |
| Worst pattern of invasion (WPOI) | Type 1 | 3 | 2 | 2 | 2.6 | 1 | 1.6 |
|  | Type2 | 24 | 16 | 14 | 18.2 | 9 | 14.3 |
|  | Type 3 | 36 | 24 | 23 | 29.9 | 11 | 17.5 |
|  | Type 4 | 60 | 40 | 29 | 37.7 | 28 | 44.4 |
|  | Type 5 | 18 | 12 | 7 | 9.1 | 10 | 15.9 |
|  | Missing/Not evaluable | 9 | 6 | 2 | 2.6 | 4 | 6.3 |
