# Supplementary material for: A combined histo-score based on tumor differentiation and lymphocytic infiltrate is a robust prognostic marker for mobile tongue cancer
Source: Virchows Arch. 2020 Jun 30;477(6):865–72. doi: 10.1007/s00428-020-02875-9 (PMC7683438; doi:10.1007/s00428-020-02875-9)
Supplement: Supplementary file 3 — (DOCX 19 kb) [file 428_2020_2875_MOESM3_ESM.docx]

| **Supplementary Table S2. Variables and categorization and disease-specific survival for the whole cohort and for low-stage and high-stage disease separately. Separate columns for resection specimens only ( biopsies excluded)** | | | | | | | |
| --- | --- | --- | --- | --- | --- | --- | --- |
|  |  |  |  |  |  |  |  |
|  |  | **Whole cohort (n=150)** | | **Low-stage (n=77)** | | **High-stage (n=63)** | |
|  |  | **All** | **Resections** | **All** | **Resections** | **All** | **Resections** |
|  | | **p (n)** | **p (n)** | **p (n)** | **p (n)** | **p (n)** | **p (n)** |
| **1.0** | **Differentiation whole tumor** | **<0.001 (126)*** | **<0.001 (106)*** | **0.003 (62)*** | **0.002 (58)*** | **0.055 (55)** | **0.035 (47)*** |
|  | Alternative 1.1 | <0.001* | <0.001* | 0.001* | 0.008* | 0.025 * | 0.035 |
| **2.0** | **Differentiation worst pattern** | **0.074 (123)** | **0.124 (106)** | **0.266 (62)** | **0.387 (58)** | **0.646 (54)** | **0.929 (47)** |
|  | Alternative 2.1 | 0.061 | 0.081 | 0.134 | 0.219 | 0.676 (54) | 0.929 |
| **3.0** | **Keratinization** | **0.030 (123)*** | **0.028 (104)*** | **0.404 (60)** | **0.584 (56)** | **0.157 (54)** | **0.065 (46)** |
|  | Alternative 3.1 | 0.007 * | 0.004* | 0.203 | 0.270 | 0.078 | 0.020* |
| **4.0** | **Keratinization tumor front** | **0.060 (119)** | **0.083 (106)** | **0.150 (61)** | **0.235 (58)** | **0.665 (52)** | **0.426 (47)** |
|  | Alternative 4.1 | 0.033* | 0.028* | 0.168 | 0.179 | 0.374 | 0.350 |
| **5.0** | **Nuclear polymorphism** | **0.176 (124)** | **0.423 (104)** | **0.005 (61)*** | **0.023 (57)*** | **0.767 (54)** | **0.514 (46)** |
|  | Alternative 5.1 | 0.109 | 0.294 | 0.021* | 0.057 | 0.673 | 0.185 |
| **6.0** | **Nuclear polymorphism tumor front** | **0.548 (119)** | **0.822 (106)** | **0.061 (61)** | **0.134 (58)** | **0.363 (52)** | **0.290 (47)** |
|  | Alternative 6.1 | 0.339 | 0.134 | 0.034* | 0.050* | 0.343 | 0.216 |
| **7.0** | **Perineural infiltration** | **0.003 (113)*** | **0.003 (101)*** | **0.263 (58)** | **0.241 (55)** | **0.046 (50)*** | **0.069 (45)** |
|  | Alternative 7.1 | 0.023* | 0.010* | 0.221 | 0.200 | 0.098 | 0.080 |
| **8.0** | **Lymphocytic infiltration** | **0.009 (122)*** | **0.029 (106)*** | **0.004 (62)*** | **0.012 (58)*** | **0.711 (54)** | **0.763 (47)** |
|  | Alternative 8.1 | 0.019* | 0.024* | 0.047* | 0.048* | 0.409 | 0.492 |
|  | Alternative 8.2 | 0.008* | 0.035* | 0.001* | 0.006* | 0.921 | 0.908 |
| **9.0** | **WPOI** | **0.218 (119)** | **0.323 (105)** | **0.248 (63)** | **0.249 (58)** | **0.997 (52)** | **0.833 (46)** |
|  | Alternative 9.1 | 0.445 | 0.362 | 0.977 | 0.605 | 0.908 | 0.719 |
|  | Alternative 9.2 | 0.027* | 0.050* | 0.069 | 0.038* | 0.902 | 0.734 |
|  |  |  |  |  |  |  |  |
|  | **T (8th)** | 0.020 (113)* | 0.005 (105)* | 0.050 (63)* | 0.019 (59)* | 0.047 (50) | 0.793 (46) |
|  | **cN0/pN0** | <0.001 (128)* | <0.001 (107)* | Only N0 | Only N0 | 0.016 (56)* | 0.115 (47) |
|  |  |  |  |  |  |  |  |
| * Significant at 0.05 level | | |  |  |  |  |  |
