# Supplementary material for: A combined histo-score based on tumor differentiation and lymphocytic infiltrate is a robust prognostic marker for mobile tongue cancer
Source: Virchows Arch. 2020 Jun 30;477(6):865–72. doi: 10.1007/s00428-020-02875-9 (PMC7683438; doi:10.1007/s00428-020-02875-9)
Supplement: Supplementary file 4 — (DOCX 17 kb) [file 428_2020_2875_MOESM4_ESM.docx]

| **Supplementary table S3. Histo-score for patients with low-stage disease. Combination of differentiation of whole tumor (1.0) and lymphocyte infiltration (8.0)** | | |
| --- | --- | --- |
|  |  |  |
| **Differentiation (score)** | **Lymphocytic infiltrate (score)** | **Sum of score (number of patients/dead*)** |
| Well (1) | Marked (1) | 2 (5/0) |
| Well (1) | Moderate (2) | 3 (10/1) |
| Moderate (2) | Marked (1) | 3 (10/0) |
| Well (1) | Slight/none (3) | 4 (4/0) |
| Moderate (2) | Moderate (2) | 4 (19/1) |
| Poor (3) | Marked (1) | 4 (0/0) |
| Moderate (2) | Slight/none (3) | 5 (9/5) |
| Poor (3) | Moderate (2) | 5 (2/1) |
| Poor (3) | Slight/none (3) | 6 (3/2) |

*Dead within 5 years after diagnosis
